# Supplementary material for: Factors associated with malnutrition inflammation score among hemodialysis patients: A cross-sectional investigation in tertiary care hospital, Palestine
Source: PLoS One. 2025 Jan 24;20(1):e0317132. doi: 10.1371/journal.pone.0317132 (PMC11761151; doi:10.1371/journal.pone.0317132)
Supplement: Attachment — Submitted filename: Point-to-point replies-MISandHD-PLOSONE.docx [file pone.0317132.s001.docx]

**Answer Letter on Review Process**

*“Factors Associated with Malnutrition Inflammation Score Among Hemodialysis Patients: A Cross-Sectional Investigation in Tertiary Care Hospital, Palestine”*

Dear editors and reviewers,

We kindly thank you for your time, effort, and time invested during the review of our manuscript. We appreciate the comments and suggestions on the submitted version of our contribution. On the basis of those, we have produced a revised and improved version, which we are submitting to PLOS ONE for an additional review.

We have formulated detailed answers to the reviewers comments, which are printed in blue as follows and highlighted in yellow in the manuscript. We hope that the answer letter and the improved version of the paper will clarify open issues, and we are looking forward to additional feedback on our revised proposal.

Kind regards,
The authors team

**Reviewer 1:**

The paper titled " Factors Associated with Malnutrition Inflammation Score Among Hemodialysis Patients: A Cross-Sectional Investigation in Tertiary Care Hospital, Palestine" need minor revision of the affiliation section, as I think it was repeated. The scientific content is good and it highlight a major cause of death in hemodialysis patients.

Thank you for your comment. Affiliations were revised and corrected.

**Reviewer 2**

1. Did all patients included in the study use the same type of membrane for hemodialysis? Were there any differences, such as the use of high-flux, low-flux, or MCO membranes? Is there any data on this? If so, could you please share it?

Thank you for your comment.

All patients in this study were dialyzed using high-flux (Fresenius) membranes (added and highlighted in yellow on the manuscript).

2. The removal of uremic solutes depends on dialysis adequacy. Expert guidelines for optimal uremic solute removal recommend a dialysis adequacy target of achieving a Kt/V urea of 1.2. Due to dialysis inadequacy, conditions such as uremic toxins and acidosis affect both protein catabolism and appetite. Is there any data on hemodialysis adequacy between the malnutrition and non-malnutrition groups? What are the Kt/V values in these two groups?

Thank you for your comment.

Currently, specific data on hemodialysis adequacy (Kt/V) are not available. However, it remains an important consideration for future studies due to its critical role in assessing solute removal efficiency and patient outcomes.

3. Of the 188 patients included in the study, 113 undergo hemodialysis twice a week. We know that as hemodialysis duration and frequency increase, better solute clearance is achieved. In the malnutrition group, were most patients undergoing hemodialysis twice a week? Was there a difference in the frequency of hemodialysis sessions between the groups?

Thank you for your comment.

We were unable to recognize the difference between nourished and malnourished groups regarding their dialysis frequency due to an incomparable data set. However, data were corrected to "179 (95.2%) are dialyzed thrice weekly" (highlighted in yellow in the manuscript), while only 4 (2.1%) are undergoing dialysis twice and 5 (2.7%) are undergoing dialysis four times weekly.

4. Is there any data on CRP, an inflammatory marker? Is there a difference in CRP levels between the malnutrition group and the non-malnutrition group? If so, could you please share it?

Thank you for your comment.

Unfortunately, data on CRP, an inflammatory marker, was not included in the collected data. We acknowledge its importance as an indicator of inflammation and its potential link to nutritional status and recommend considering CRP analysis in future research.

However, we can consider ferritin, which is an acute-phase reactant, as an inflammatory marker that showed a significant association in our findings, as shown in the table below:

| Biochemical indices  (Mean ± SD) | Total (SI unit)  N = 188 | Malnutrition | | P-value |
| --- | --- | --- | --- | --- |
|  |  | Yes | No |  |
| Ferritin ng/ml | 714.4 ± 372.3 (1605.26 pmol/l) | 896.8 ± 411.1 | 644 ± 331.5 | <0.001* |

Accordingly, the following paragraph has been added to the discussion (highlighted in yellow in the manuscript):

“Interestingly, serum ferritin is widely recognized as an acute-phase reactant, frequently elevated in CKD patients as a result of systematic inflammation, and correlates positively with the severity of inflammation [R1,R2].”

In addition, the following paragraph has been added as a limitation and recommendation for future studies (highlighted in yellow in the manuscript):

“Moreover, insufficient data on C-reactive protein (CRP) and dialysis adequacy limit the capacity to assess their potential link to nutritional status among HD patients in this study. Including such information could provide significant insights and should be considered for future investigations.”

[R1]: Tomasz G, Ewa W, Jolanta M. Biomarkers of iron metabolism in chronic kidney disease. Int Urol Nephrol. 2021;53: 935–944. doi:10.1007/s11255-020-02663-z

[R2]: Fu S, Chen J, Liu B, Liang P, Zeng Y, Feng M, et al. Systemic inflammation modulates the ability of serum ferritin to predict all-cause and cardiovascular mortality in peritoneal dialysis patients. BMC Nephrol. 2020;21: 1–9. doi:10.1186/s12882-020-01892-9

5. For HD patients, dietary energy intake (DEI) and dietary protein intake (DPI) recommendations for adults have been proposed by various expert groups. These generally range from 25–35 kcal/kg ideal body weight (IBW)/day for DEI and 1.0–1.2 g protein/kg IBW/day for DPI. What is your definition of inadequate nutrient intake?

Thank you for your comment.

Inadequate nutrient intake refers to dietary energy and protein intake levels that fail to meet established recommendations necessary for maintaining nutritional balance and health. Among HD patients, inadequate nutrient intake is when their energy intake is below 25 kcal/kg/day and their protein intake is lower than 1.0 g/kg/day [R3]. However, the following paragraph has been added, and calorie and protein requirements have been edited in Table 2 (highlighted in yellow in the manuscript):

“The nutritional intake of study participants is considered inadequate when their energy intake is below 25 kcal/kg/day and their protein intake is lower than 1.0 g/kg/day [R1].”

[R3]: Diaz-Martinez J, Bejar C, Delgado-Enciso I. The relationship of nutrition status and dietary intake with hospitalization and mortality in hemodialysis patients; a single-center observational cohort study. J Nephropathol. 2023;12. doi:10.34172/jnp.2022.17301

6. The dialysis process leads to chronic nutrient losses, particularly of proteins and amino acids. Hypoalbuminemia is a strong predictor of malnutrition. According to your study, a lower albumin level was found to reduce the risk of developing malnutrition. How can you explain this?

Thank you for your comment.

In our findings, the univariate analysis showed a significant association between hypoalbuminemia and increased malnutrition, but in the multivariate analysis, hypoalbuminemia was found to reduce the risk of malnutrition, which is counterintuitive. This could be explained by confounding variables, which might influence the association and mask the link between low albumin and malnutrition. In addition, selection bias could be the reason for such a result in which healthier patients with mild hypoalbuminemia might have been included in this study. Otherwise, patients with lower albumin levels might be receiving more aggressive nutritional support, thus improving their nutritional status. However, more studies are recommended in this regard for a comprehensive understanding.

The following paragraph has been added in light of your note (highlighted in yellow in the manuscript):

“Nevertheless, further explanations for our findings regarding the protective effect of low albumin levels against malnutrition may include confounding factors, which might influence the association and mask the link between low albumin and malnutrition. In addition, this finding could be explained by selection bias, in which healthier patients with mild hypoalbuminemia might be included in this study.”
